# The current state of mental healthcare in Bangladesh: part 1 – an updated country profile

**DOI:** 10.1192/bji.2021.41

**Published:** 2021-11

**Authors:** M. Tasdik Hasan, Tasnim Anwar, Enryka Christopher, Sahadat Hossain, Md Mahbub Hossain, Kamrun Nahar Koly, K. M. Saif-Ur-Rahman, Helal Uddin Ahmed, Nazish Arman, Saima Wazed Hossain

**Affiliations:** 1 Consultant (Mental Health), Shuchona Foundation, Dhaka, Bangladesh. Email: tasdik.hasan@liverpool.ac.uk; 2 Graduate Student, Kings College London, UK; 3 Center for Population and Development Studies, Harvard T.H. Chan School of Public Health, Cambridge, MA, USA; 4 Lecturer, Department of Public Health & Informatics, Jahangirnagar University, Savar, Dhaka, Bangladesh; 5 DrPH Researcher, Department of Health Promotion and Community Health Sciences, Texas A&M School of Public Health, College Station, TX, USA; 6 Assistant Scientist, Health Systems and Population Studies Division, icddr,b, Dhaka, Bangladesh; 7 Associate Professor, Child & Adolescent Psychaitry, National Institute of Mental Health, Sher-e-Bangla Nagar, Dhaka, Bangladesh; 8 Lead Coordinator for Content Development, Shuchona Foundation, Dhaka, Bangladesh; 9 Chairperson, Shuchona Foundation, Dhaka, Bangladesh

**Keywords:** Mental health, mental health system, mental health policy, priorities, Bangladesh

## Abstract

Mental health is a significant factor for a sound and productive life; nevertheless, mental disorders do not often receive adequate research attention and are not addressed as a serious public health issue in countries such as Bangladesh. Part 1 of this two-part profile describes the current situation of mental health in Bangladesh in its wider sociocultural context, outlining existing policies and highlighting mental illness as a neglected healthcare problem in the country using a narrative synthesis method. The prevalence of mental disorders is very high and augmented in nature among different population groups in Bangladesh. A lack of public mental health facilities, scarcity of skilled mental health professionals, insufficient financial resource distribution, inadequately stewarded mental health policies and stigma contribute to making current mental healthcare significantly inadequate in Bangladesh. The country has few community care facilities for psychiatric patients. Furthermore, the current mental health expenditure by the Bangladeshi government is only 0.44% of the total health budget. Less than 0.11% of the population has access to free essential psychotropic medications.

## Background

Bangladesh, a lower middle-income country in South Asia, has a population of 163 million, making it the world's eighth most populous country.^1,2^ Two-thirds of the population reside in rural areas. Literacy rates are estimated at 75.62% for males and 69.90% for females.^3^ With only 4 hospital beds per 10 000 people, Bangladesh faces an immense burden of illness arising from both communicable and non-communicable diseases, including mental disorders.^4,5^ Mental healthcare in Bangladesh is enormously inadequate owing to a lack of public mental health facilities, scarcity of skilled mental health professionals, insufficient financial resource distribution and societal stigma. These shortcomings are sustained by the absence of effective stewardship to execute adequate mental health policies.^5^

Despite limited documentation of the burden of mental disorders and challenges in improving mental healthcare, there has been no comprehensive review of the country's current mental health state at the national level. In part 1 of this country profile we will (a) summarise the current mental health evidence based on a literature review, (b) describe the current situation of mental disorders in Bangladesh in its wider sociocultural context and (c) chronicle the existing mental healthcare services and financing in the country.

## Current context and vulnerable groups

### Adults

The first national survey on mental health in Bangladesh was conducted in 2003–2005.^5^ The second (and most recent) nationwide representative survey was conducted in 2019 (Table 1).^6^

**Table 1:** Prevalence of all mental disorders among the adult population in Bangladesh^5–7^

	1974	2005	2019
All adults	31.4%	16.1%	18.7%
Men	Not reported	12.9%	12.5%
Women	Not reported	19.0%	18.8%

The coronavirus disease 2019 (COVID-19) pandemic has wreaked havoc on the mental health of the Bangladeshi population. One study has found that the prevalence of depressive (57.9%), stress (59.7%) and anxiety (33.7%) symptoms in the adult population is now much higher than pre-pandemic rates.^8^ Another study found that 28.5%, 33.3% and 46.92% of home-quarantined students had stress, anxiety and depressive symptoms respectively.^9^

### Children

A systematic review of literature on children in Bangladesh from 1998 to 2004 found prevalence estimates of mental disorders between 13.4 and 22.9%.^7^ Community-based surveys in 2004 and 2009 report prevalence estimates in line with those of past decades. The most recent figures were obtained from the 2019 nationwide representative survey, and report a lower prevalence for girls than boys (Table 2).^6^

**Table 2:** Prevalence of all mental disorders among children in Bangladesh^6,7,10,11^

	2004	2009	2019
All children	15.0%	18.4%	12.6%
Boys	Not reported	Not reported	13.7%
Girls	Not reported	Not reported	11.5%

### Other child and adolescent groups

A systematic review estimated the prevalence of autism spectrum disorders as between 0.2 and 0.8% in Bangladesh.^10^ A 2013 study reported that 25% of adolescents in urban schools experienced depressive symptoms (girls: 30%, boys: 19%).^11^ A 2018 study among adolescents in urban and semi-urban schools found that 36.6% suffered from depressive symptoms (girls: 42.9%, boys: 25.7%).^12^ A similar prevalence of depression (38.9%) was reported among Bangladeshi medical students in 2013.^13^ A 2019 study among university students reported a 22.5% increase in the prevalence of depression (meeting provisional diagnostic criteria) and a 27.1% increase in the prevalence of anxiety within a 15-month period.^14^

## Mental healthcare delivery, services and systems

Of approximately 7000 graduates each year from medical schools across the country, only a few choose to specialise in psychiatry.^15^

Mental health services are provided by psychiatrists, psychiatric nurses and clinical psychologists, with little to no multidisciplinary teamwork between them. It is difficult for rural populations to access psychiatrists and other mental health professionals.^16^ Mental health services are often limited to a divisional tertiary level, where psychiatrists work at public medical college hospitals located within cities. With only 260 psychiatrists serving a country of 162 million, much of the population is unable to access mental health services.^17^

The few community care facilities for psychiatric patients available throughout the country are greatly strained in terms of both human and financial resources. There is currently only one national-level mental health institute in the country, the National Institute of Mental Health (NIMH), in Dhaka.^18^ A 200-bed mental health hospital comprises part of the NIMH, with an additional 500-bed psychiatric hospital located nearby.^16^ An additional 15 beds exist in forensic in-patient units and 3900 beds in residential facilities, including homes for the destitute, in-patient detoxification centres and homes for people with severe neurodevelopmental disorders. A few substance misuse treatment and rehabilitation facilities are organised by private practitioners and unregulated. At the community level, 31 psychiatric in-patient units exist,^16^ which account for only 8% of the total number of hospital beds in the country.^5^ Mentally ill persons constitute 4.2% of the patients served in in-patient units, suggesting possible reluctance of health professionals to assign in-patient stays for mental illness.^5^ The average length of stay for a mental health in-patient is 137 days.

The first out-patient clinic for mental illness was established by the Dhaka Medical College and Hospital in 1969. There are currently 50 out-patient mental health facilities in the country, but the majority of them are located in urban areas.^15^ Some non-governmental organisations (NGOs) also contribute to the provision of mental healthcare in Bangladesh. Although the NIMH began to provide mental health training to primary care physicians and health workers in 1981, community-based follow-up is limited, especially for those in rural areas.

The limited knowledge about mental health in Bangladesh contributes to a lack of sufficient care programmes, thereby neglecting the mental health needs of the population. Unfortunately, few NGOs cater to improving mental health. When disasters strike, mental health is overlooked by authorities, evidenced by the absence of attention given to disaster-related mental disorders such as post-traumatic stress disorder (PTSD) in Bangladeshi clinical and policy fields. The term PTSD was first brought to public attention in relation to war veterans, but this disorder can result from a variety of traumatic incidents, such as muggings, rape, torture, being kidnapped or held captive, childhood abuse, car accidents, train wrecks, plane crashes, bombings and natural disasters, including floods and earthquakes.^19^ PTSD is not emphasised as an important health concern in the healthcare system of Bangladesh, begetting a fundamental gap in mental healthcare, given the country's proneness to natural disasters owing to its geography and the frequency of man-made disasters, such as building collapses and urban fires. In 2017, the Mental Health Gap Action Programme (mhGAP) was implemented with relative success by the government of Bangladesh to address the humanitarian crisis at Cox's Bazar.^20^ However, this programme has yet to be scaled up to the rest of the nation.

Although antipsychotics, anxiolytics, antidepressants, mood stabilisers and anti-epileptic drugs are included in the list of essential medicines recommended by the WHO, psychotropic drugs are not widely available in Bangladesh.^21,22^ Only a few patients visiting the government healthcare facilities have access to these psychotropic medications. Despite the well-structured three-tier healthcare delivery system in Bangladesh,^23^ a lack of qualified mental healthcare professionals and limited logistical support lead to a discrepancy in meeting the mental healthcare needs of the population.^17^ Studies have highlighted low levels of help-seeking as well as poor service delivery for mental health conditions in Bangladesh.^7^

Referrals of patients with mental illness to mental health specialists by primary care physicians or other healthcare providers are near non-existent.^24^ Superstitious beliefs regarding the causation of psychiatric disorders prevent help-seeking from mental health services. Psychiatric disorders, including psychotic disorders, are commonly perceived as being triggered by supernatural influences, with the cure often sought from traditional healers. Although these traditional practices have been shown to benefit outcomes for some mental health conditions, these practices show little to no benefit for psychotic illness and are a wasteful economic cost to the majority low-income population. Some practices may also be physically and psychologically harmful, further complicating prognosis.^7^

## Financing of mental health services

Mental health expenditures by the Bangladeshi government are 0.44% of the total health budget.^25^ Of all the expenditure on mental health, 67% is dedicated to mental hospitals. Less than 0.11% of the population have access to free essential psychotropic medications. Daily out-of-pocket expenses for the lowest-priced antipsychotic and antidepressant medication is 5.00 taka (US$ 0.07) and 3.00 taka (US$ 0.04) respectively. Health insurance is a rarity and, in any case, typically does not cover drugs for mental illness.^5^

## Human resources for mental healthcare

Very few healthcare workers in Bangladesh are trained in providing mental health services (0.49%), and there are even fewer psychiatrists (0.16 per 100,000 population).^17^ The majority of these professionals work in the urban areas of the country, namely the capital city of Dhaka. Types of mental healthcare providers in Bangladesh include psychiatrists, nurses, psychologists, social workers, occupational therapists and general mental health workers.^5,15^ Approximately half the psychiatrists (54%) in Bangladesh work in government mental health facilities or private sector clinics; 46% work for NGOs, for-profit mental health facilities or in private practice.^5^ Psychiatrists working in government facilities are allowed to concurrently work in the private sector as well. Around 62% of psychosocial professionals, including clinical psychologists, social workers, nurses and occupational therapists, work for government-administered mental health facilities, 26% work for NGOs or in private practice, and 12% work for both the public and the private sectors. The distribution of human resources between urban and rural areas is grossly disproportionate, with a heavy concentration in urban areas.^26–28^ The density of psychiatrists and psychiatric nurses in or around the largest city, the capital Dhaka, is five times greater than the density of these professionals in the rest of the country.^5^ Bangladesh's available workforce in mental healthcare is scarce and skewed in distribution, an immense barrier to improving mental healthcare in the country.

## Social stigma, inequalities and sociocultural influences

High social stigma attached to mental illness also affects help-seeking behaviour. Consequently, mentally ill persons suffer in silence, with social isolation and discrimination. Morbidity from psychiatric illness remains high and is seldom regarded as a public health concern. Widespread stigma towards the mentally ill in Bangladesh is attributable to superstitions surrounding causation of mental illness. Mental disorder is perceived to be a consequence of possession by evil spirits, as opposed to biological or psychological mechanisms, leading to neglect and abuse of those with mental illness.^29^

Other mental health issues pertinent to Bangladesh include gender-based violence and substance misuse. Approximately 60% of ever-married women in Bangladesh reported experiences of sexual and physical intimate partner violence, a matter that remains largely ignored by the government and policymakers. Very little is known about violence against unmarried female adolescents.^30^ Domestic violence, dowry-related acid attacks, rape, forced abortion and trafficking for prostitution are common gender-based violence problems, with victims often facing severe psychological and psychosomatic symptoms.^31^

The perceived physical health of the population is another important predictor of mental disorder in Bangladesh. A recent study among university students revealed a significant association between students’ self-perceived physical health conditions and symptoms of depression and anxiety.^32^ Additionally, a growing national concern is substance misuse, which has increased in recent years among young women.^33^ Drug use is highest among Bangladeshi young people between the ages of 15 and 30 years. Despite the proven mental health consequences arising from substance misuse, Bangladesh has limited services available to rehabilitate this population. There are only a few public, private sector or NGO-run facilities for those struggling with substance misuse. Moreover, these services are primarily located in urban areas, leaving a vast majority of the population devoid of such services.^5^

## Conclusions

The prevalence of mental disorders in Bangladesh is very high, and treatment is neglected, especially among marginalised populations. A lack of public mental health facilities, scarcity of skilled mental health professionals, insufficient financial resource distribution and stigma contribute to the barriers to accessing mental healthcare in Bangladesh. At a macro level, lack of healthcare expenditure by the Bangladeshi government, poor advocacy and limited research further exacerbate the problem. In part 2 of this profile we suggest priorities for improving the nation's mental health.

## Data Availability

Data availability is not applicable to this article as no new data were created or analysed in this study.

## References

[1] World Bank. Bangladesh - World Bank Open Data. World Bank Group, 2019 (https://data.worldbank.org/country/bangladesh [cited 19 Dec 2020]).

[2] Martin E. Goldman Sachs's MIST topping BRICs as smaller markets outperform. *Bloomberg News*, 2012 (https://www.bloomberg.com/news/articles/2012-08-07/goldman-sachs-s-mist-topping-brics-as-smaller-markets-outperform [cited 19 Dec 2020]).

[3] UNESCO. Bangladesh. UNESCO, 2018 (https://en.unesco.org/countries/bangladesh [cited 19 Dec 2020]).

[4] Siddiqui MMR, Rahman F, Islam SMR-u. Bangladesh is experiencing double burden with infectious diseases and non-communicable diseases (NCD's): an issue of emerging epidemics. Anwer Khan Mod Med Coll J 2014; 5: 46–50.

[5] Islam A, Biswas T. Mental health and the health system in Bangladesh: situation analysis of a neglected domain. Am J Psychiatry Neurosci 2015; 3(4): 57–62

[6] National Institute of Mental Health. National Mental Health Survey of Bangladesh 2018–19: Provisional Fact Sheet. NIMH, 2019 (https://www.who.int/docs/default-source/searo/bangladesh/pdf-reports/cat-2/nimh-fact-sheet-5-11-19.pdf?sfvrsn=3e62d4b0_2 [cited 16 Dec 2020]).

[7] Hossain MD, Ahmed HU, Chowdhury WA, Niessen LW, Alam DS. Mental disorders in Bangladesh: a systematic review. BMC Psychiatry 2014; 14: 216. 25073970 10.1186/s12888-014-0216-9 PMC4149198

[8] Banna MHA, Sayeed A, Kundu S, Christopher E, Hasan MT, Begum MR, The impact of the COVID-19 pandemic on the mental health of the adult population in Bangladesh: a nationwide cross-sectional study. Int J Environ Health Res [Epub ahead of print] 2 Aug 2020. Available from: 10.1080/09603123.2020.1802409. 32741205

[9] Khan AH, Sultana MS, Hossain S, Hasan MT, Ahmed HU, Sikder MT. The impact of COVID-19 pandemic on mental health & wellbeing among home-quarantined Bangladeshi students: a cross-sectional pilot study. J Affect Disord 2020; 277: 121–8. 32818775 10.1016/j.jad.2020.07.135 PMC7410816

[10] Hossain MD, Ahmed HU, Jalal Uddin MM, Chowdhury WA, Iqbal MS, Kabir RI, Autism spectrum disorders (ASD) in South Asia: a systematic review. BMC Psychiatry 2017; 17(1): 281. 28826398 10.1186/s12888-017-1440-x PMC5563911

[11] Khan A, Ahmed R, Burton NW. Prevalence and correlates of depressive symptoms in secondary school children in Dhaka city, Bangladesh. Ethn Health 2020; 25: 34–46. 29096523 10.1080/13557858.2017.1398313

[12] Anjum A, Hossain S, Sikder T, Uddin ME, Rahim DA. Investigating the prevalence of and factors associated with depressive symptoms among urban and semi-urban school adolescents in Bangladesh: a pilot study. Int Health [Epub ahead of print] 6 Nov 2019. Available from: 10.1093/inthealth/ihz092. PMC10575608 31693088

[13] Hasan MT, Hossain S, Gupta RD, Podder V, Afroz Mowri N, Ghosh A, . Depression, sleeping pattern, and suicidal ideation among medical students in Bangladesh: a cross-sectional pilot study. J Public Health 1 Jun 2020. Available from: 10.1007/s10389-020-01304-0.

[14] Hossain S, Anjum A, Uddin ME, Rahman MA, Hossain MF. Impacts of socio-cultural environment and lifestyle factors on the psychological health of university students in Bangladesh: a longitudinal study. J Affect Disord 2019; 256: 393–403. 31226611 10.1016/j.jad.2019.06.001

[15] World Health Organization. WHO-AIMS Report on Mental Health System in Bangladesh. WHO, 2007 (http://www.who.int/mental_health/bangladesh_who_aims_report.pdf [cited 19 Dec 2020]).

[16] Nuri NN, Sarker M, Ahmed HU, Hossain MD, Beiersmann C, Jahn A. Pathways to care of patients with mental health problems in Bangladesh. Int J Ment Health Syst 2018; 12: 39. 30034515 10.1186/s13033-018-0218-y PMC6052552

[17] World Health Organization. Bangladesh WHO Special Initiative for Mental Health Situational Assessment. WHO, 2020 (https://cdn.who.int/media/docs/default-source/mental-health/special-initiative/who-special-initiative-country-report---bangladesh---2020_f746e0ca-8099-4d00-b126-fa338a06ca6e.pdf?sfvrsn=c2122a0e_7 [cited 10 Aug 2021]).

[18] Choudhury WA, Quraishi FA, Haque Z. Mental health and psychosocial aspects of disaster preparedness in Bangladesh. Int Rev Psychiatry 2006; 18: 529–35. 17162693 10.1080/09540260601037896

[19] National Institute of Mental Health. Post-Traumatic Stress Disorder. NIMH, 2019 (https://www.nimh.nih.gov/health/topics/post-traumatic-stress-disorder-ptsd/index.shtml [cited 19 Dec 2020]).

[20] Momotaz H, Ahmed H, Jalal Uddin MM, Karim R, Khan MA, Al-Amin R, . Implementing the Mental Health Gap Action Programme in Cox's Bazar, Bangladesh. Intervention 2019; 17: 243–51.

[21] Baumann P, Hiemke C, Ulrich S, . The AGNP-TDM expert group consensus guidelines: therapeutic drug monitoring in psychiatry. Pharmacopsychiatry 2004; 37(6): 243–65. 15551191 10.1055/s-2004-832687

[22] Reich MR. Bangladesh pharmaceutical policy and politics. Health Policy Plan 1994; 9: 130–43. 15726775 10.1093/heapol/9.2.130

[23] Alam D, Robinson H, Kanungo A, Hossain MD, Hassan M. Health Systems Preparedness for Responding to the Growing Burden of Non-Communicable Disease – A Case Study of Bangladesh (Working Paper 25, Health Health Policy & Health Finance Hub). University of Melbourne/Nossal Institute for Global Health, 2013 (https://www.researchgate.net/publication/262451665 [cited 19 Dec 2020]).

[24] Chowdhury AK, Salim M, Sakeb N. Some aspects of psychiatric morbidity in the out-patient population of a general hospital. Bangladesh Med Res Counc Bull 1975; 1: 51–9. 1244015

[25] Alam F, Hossain R, Ahmed H, Alam M, Sarkar M, Halbreich U. Stressors and mental health in Bangladesh: Current situation and future hopes. BJPsych Int 2020: 1–4. Available from: 10.1192/bji.2020.57 34747936 PMC8554941

[26] Islam A, Biswas T. Health system in Bangladesh: challenges and opportunities. Am J Health Res 2014; 2: 366–74

[27] Islam A, Biswas T. Chronic non-communicable diseases and the healthcare system in Bangladesh: current status and way forward. Chronic Dis Int 2014; 1(2): 6.

[28] Islam A, Biswas T. Health system bottlenecks in achieving maternal and child health-related millennium development goals: major findings from district level in Bangladesh. J US-China Med Sci 2014; 11: 147–58.

[29] Adams AM, Ahmed T, El Arifeen S, Evans TG, Huda T, Reichenbach L. Innovation for universal health coverage in Bangladesh: a call to action. Lancet 2013; 382: 2104–11. 24268605 10.1016/S0140-6736(13)62150-9

[30] Naved RT, Rimi NA, Jahan S, Lindmark G. Paramedic-conducted mental health counselling for abused women in rural Bangladesh: an evaluation from the perspective of participants. J Health Popul Nutr 2009; 27: 477–91. 19761082 10.3329/jhpn.v27i4.3391 PMC2928104

[31] Hossain KT, Sumon MSR. Violence against women: nature, causes and dimensions in contemporary Bangladesh. Bangladesh e-J Sociol 2013; 10: 79–91.

[32] Hossain S, Anjum A, Hasan MT, Uddin ME, Hossain MS, Sikder MT. Self-perception of physical health conditions and its association with depression and anxiety among Bangladeshi university students. J Affect Disord 2020; 263: 282–8. 31818790 10.1016/j.jad.2019.11.153

[33] Hossain S, Hossain S, Ahmed F, Islam R, Sikder T, Rahman A. Prevalence of tobacco smoking and factors associated with the initiation of smoking among university students in Dhaka, Bangladesh. Centr Asian J Glob Health 2017; 6(1): 244. 10.5195/cajgh.2017.244 PMC5675390 29138736

[34] Hasan MT, Anwar T, Christopher E, Hossain S, Hossain MM, Koly KN, The current state of mental healthcare in Bangladesh: part 2 – setting priorities. BJPsych Int 2021; 18: 82–85. 10.1192/bji.2021.42 PMC8554892 34747940

